# Supplementary material for: Utility of Physiologically Based Pharmacokinetic Modeling to Investigate the Impact of Physiological Changes of Pregnancy and Cancer on Oncology Drug Pharmacokinetics
Source: Pharmaceutics. 2023 Dec 4;15(12):2727. doi: 10.3390/pharmaceutics15122727 (PMC10748010; doi:10.3390/pharmaceutics15122727)
Supplement: Supplementary file 1 [file pharmaceutics-15-02727-s001.zip › pharmaceutics-2549194-supplementary.pdf]

Utility of Physiologically Based Pharmacokinetic Modeling to Investigate the Impact of Physiological Changes of Pregnancy and Cancer on Oncology Drug Pharmacokinetics

Xinxin Yang, Manuela Grimstein, Michelle Pressly, Elimika Pfuma Fletcher, Stacy Shord and Ruby Leong *

Office of Clinical Pharmacology, Center for Drug Evaluation and Research, U.S. Food and Drug Administration, 10903 New Hampshire Avenue, Silver Spring, MD 20993, USA; xinxin.yang@bms.com (X.Y.); manuela.grimstein@fda.hhs.gov (M.G.); stacy.shord@fda.hhs.gov (S.S.)

* Correspondence: ruby.leong@fda.hhs.gov; Tel.: +1-240-401-3064

Supplemental Materials

Drug Model Development and Verification

1. Paclitaxel

1.1. Drug model

The PBPK model of paclitaxel was adopted from Mendes et al. [1]. Input parameters are summarized in **Table S1**. Three different drug models are available for paclitaxel to characterize the dose dependent effects on paclitaxel plasma protein binding, volume of distribution and drug clearance [1]. This PBPK analysis used and focused on validating the paclitaxel drug model at 175 mg/m^2 as this dosage was reported in clinical studies with pregnant cancer patients [2, 3]. The compound file was developed for IV administration with full PBPK distribution model. The distribution to all organs was assumed to be perfusion-limited, except the hepatic distribution was described by a permeability limited liver model to allow for biliary excretion of paclitaxel mediated by P-glycoprotein (P-gp) in the model. Rodgers and Rowland method with addition of membrane potential (method 3 in Simcyp V21) were selected to predict the volume of distribution at steady-state (Vss). The Kp Scalar were adjusted to 0.14 to set the Vss as 0.9 L/kg. Paclitaxel was mainly cleared by metabolic, renal and biliary routes based on urinary and fecal recovery of paclitaxel [4]. The calculated CYP2C8 and CYP3A4 intrinsic clearance (CL_{int}) values were determined as 3.06 and 0.54 µL/min/pmol, respectively, to recover the fraction metabolized (fm) values of approximately 50% for both enzymes. A biliary CL_{int} value of 3.91 µL/min/million cells was then calculated using the retrograde method by accounting 7% of the total clearance to the biliary route and assigned to the canalicular efflux mediated by P-gp. Additionally, average renal clearance of 1.75 L/h was reported in cancer patients [4]. This value was corrected by the GFR difference between cancer population and a healthy subject (age of 20-30 years) to use as input. The renal clearance was assumed to be constant across doses. The mean contribution of each elimination pathway to total paclitaxel clearance for the dose of 175 mg/m^2 is as follows: around 43%, 37%, 13% and 7% for CYP3A4, CYP2C8, renal elimination and biliary clearance, respectively.

Table S1. **Physicochemical and pharmacokinetic input parameters for the PBPK model of paclitaxel**

Parameter	Value	Reference
**Molecular weight (g/mol)**	853.9	Mendes et al. (2020) [1]
**log P**	3.54	
**Compound type**	Neutral	
**B/P**	0.69	
**fu**	0.054	
**Main plasma binding protein**	Human serum albumin	
Distribution Model	Full PBPK Model	
**VSS (L/kg)**	0.909	
**Kp scalar**	0.14	
Elimination		
**Enzyme CYP2C8 CLint (μL/min/pmol)**	3.06	
**Enzyme CYP3A4 CLint (μL/min/pmol)**	0.536	
**CLR (L/h)**	2.658	
**CLPD (mL/min/million hepatocytes)**	0.6	
**Transporter ABCB1 (P-gp/MDR1) CLint,T (μL/min/million cells)**	3.91	

Kp scalar: Scalar applied to all predicted tissue kp (tissue:plasma partition coefficients) value;
CLPD: passive diffusion clearance

1.2 Model validation

The drug model predictive performance for PK was evaluated by comparing the predicted PK parameters with the observed values reported in Villalona-Calero et al., Kendra et al. and Brouwer et al. [5-7]. In addition, the contribution of CYP3A4 and CYP2C8 to paclitaxel clearance in the model was verified by the comparison of observed vs predicted interaction effect (AUC ratio) of verapamil (CYP3A4 and P-gp inhibitor) and pazopanib (CYP3A4 and CYP2C8 inhibitor) on the PK of paclitaxel [1, 6, 8]. The detailed simulation condition for each study can be found in **Table S2**.

Table S2. Trial design for paclitaxel model simulations

Drug	Design	Reference (Observation)
Paclitaxel	Ten virtual trials with 8 subjects aged 48-72 years (24% female) receiving a single dose of 17 5mg/m2 paclitaxel infused over 3 hours.	Villalona-Calero et al. (1999) [5]
	Ten virtual trials with 4 subjects aged 31-80 years (46% female) receiving a single dose of 175 mg/m2 paclitaxel infused over 3 hours.	Kendra et al. (2015) [6]

	Ten virtual trials with 3 subjects aged 51-64 years (66% female) receiving a single dose of 175 mg/m2 paclitaxel infused over 3 hours.	Brouwer et al. (2000) [7]
	Ten virtual trials with 6 subjects aged 30-76 years (100% female) were generated. Each subject received a single dose of 200mg/m2 paclitaxel infused over 3 hours in the absence or presence of verapamil at 225 mg/m2 every 4 hours for 12 doses beginning 24 hours before the start of paclitaxel infusion.	Berg et al. (1995) [8]
	Ten virtual trials with 6 subjects aged 31 to 80 years (46% female) receiving a a single dose of 175 mg/m2 paclitaxel infused over 3 hours in the absence of pazopanib and on the last day of 21 days of dosing with pazopanib (400 mg QD)	Kendra et al. (2015) [6]
	Ten virtual trials with 4 subjects aged 31 to 80 years (46% female) receiving a a single dose of 175 mg/m2 paclitaxel infused over 3 hours in the absence of pazopanib and on the last day of 21 days of dosing with pazopanib (800 mg QD)	

1.3 Results

The model predicted geometric mean values for Cmax, AUCinf and CL of paclitaxel (175 mg/m^2) were 3.77 µg/ml, 17.5 µg/ml*h and 18.2 L/h respectively. The predictions compared favorably with the observed geometric mean values for $C_{max,}$ AUCinf and CL of 3.98 µg/ml, 16.0 µg/ml*h and 21.2 L/h, respectively, reported in Villalona-Calero et al. [5]. The model adequately predicted the PK of paclitaxel reported in Kendra et al. (**Table S3a**); however, no time-concentration data was reported in this study to allow comparison of the PK profile. In addition, the model adequately predicted the PK of paclitaxel reported in Brouwer et al. (**Table S3a**). The predicted and observed paclitaxel plasma concentration-time profile after a single 3-hour IV infusion of 175 mg/m^2 are shown in **Figure S1**.

The relative contribution of CYP3A4 and CYP2C8 to paclitaxel metabolism was adequately assigned in the model as verified by the clinical DDI data with strong CYP3A (verapamil) and CYP2C8 (pazopanib) inhibitor. In the presence of verapamil 225 mg/m^2 q4h for 48 hours, the predicted vs observed changes in the AUC ratio were 1.51-fold vs 1.76-fold. In the presence of pazopanib 400 mg or 800 mg QD for 21 days, the predicted vs observed changes in the AUC ratio were 1.11-fold vs 1.10-fold and 1.15-fold vs 1.30-fold, respectively (**Table S3b**).

Table S3a. Comparison of clinically observed and PBPK predicted PK parameters of paclitaxel

**Paclitaxel**		**C_{max} (µg/ml)**	**AUC_{inf} (µg/ml*h)**	**CL (L/h)**
**Villalona-Calero et al. (1999) [5]**	Predicted (n=80)	3.77 ± 0.98	17.48 ± 6.3	18.15 ±5.97
		2.84-5.33	11.45-29.62	10.77-29.06
**175 mg/m^2 3-hour infusion**	Observed (n=8)	3.98 ± 1.39	16.0 ±3.37	21.2 ± 4.0

**PK parameters reported as geometric mean ± SD, 95% CI**		1.2-6.76	9.3-22.8	13.2-29.2
	**Pred/Obs**	**0.95**	**1.09**	**0.86**
**Kendra et al. (2015) [6]**	Predicted (n=40)	3.58	15.61	20.63
**175 mg/m^2 3-hour infusion**		2.42-6.13	9.02-34.7	9.55-41.58
	Observed (n=4)	5.02	16	22.3
**PK parameters reported as median and range**		3.30-6.25	10.9-19.7	20.3-39.1
	**Pred/Obs**	**0.71**	**0.98**	**0.93**
**Brouwer et al. (2000) [7]**	Predicted (n=30)	3.98	17.79	20.7
**175 mg/m^2 3-hour infusion**	Observed (n=3)	3.58	15.5	22.8
**PK parameters reported as mean Cmax (µM), AUC (µMh)**	**Pred/Obs**	**1.11**	**1.15**	**0.91**

Table S3b. Observed and predicted PK parameters ratios for paclitaxel in absence and presence of R-Verapamil (CYP3A4 and P-gp inhibitor) and pazopanib (CYP3A4 and CYP2C8 inhibitor)

Study	Inhibitor		Cmax Ratio	AUC Ratio	CL Ratio
**Berg et al. (1995) [8]**	**Verapamil 225mg/m^2 (n=6)**	Predicted		1.51	0.66
		Observed	N/A	1.76	0.56
**PK parameters reported as median**		**Pred/Obs**		**0.86**	**1.19**
**Kendra et al. (2015) [6]**	**Pazopanib (400 mg) (n=6)**	Predicted	1.05	1.10	0.91
		Observed	1.02	1.10	0.86
		**Pred/Obs**	**1.02**	**1.00**	**1.06**
**PK parameters reported as mean for 400 mg dose, median for 800 mg dose**	**Pazopanib (800 mg) (n=4)**	Predicted	1.08	1.18	0.86
		Observed	1.31	1.30	0.72
		**Pred/Obs**	**0.82**	**0.91**	**1.19**

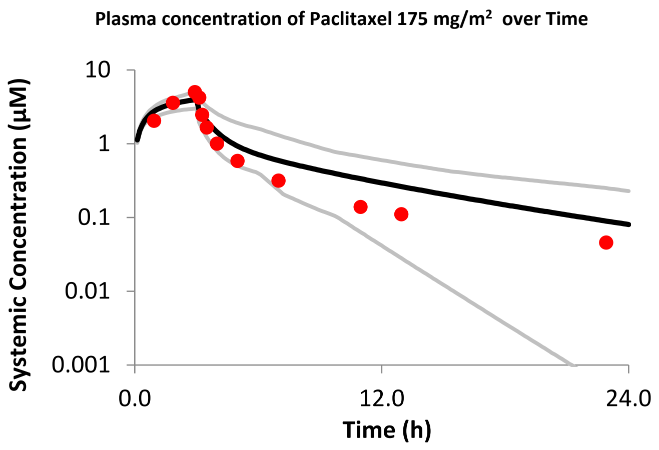

Figure S1. PBPK predicted vs observed (Brouwer et al. [7]) plasma concentration-time profiles of paclitaxel in nonpregnant cancer patients after 3-h infusion of 175 mg/m^2 dose. Red circles represent the mean observed PK profile. Black and grey lines represent the population mean and 5th-95th percentiles of the predicted PK profile, respectively.

2 Docetaxel

2.2 Model development

A new drug model for docetaxel was developed based on a previous published model [9]. Input parameters and PBPK model development for docetaxel are summarized in **Table S4**. The molecular weight, partition coefficient (LogP) and blood-to-plasma ratio values were obtained from literature [9]. Docetaxel is significantly bound to plasma proteins (fu= 0.06) in cancer patients [10]. The fraction unbound (fu) was predicted by considering the in vitro binding affinity of docetaxel to alpha-1-acid glycoprotein (AAG), albumin, and lipoprotein, [11], as well as plasma concentrations of AAG and albumin in cancer patients, implemented in the default cancer population model. A full PBPK distribution model was selected and Vss was predicted using the Rodgers and Rowland method (Method 2 in SimCYP V21). The systemic clearance of docetaxel (CL_{IV}) was reported to be around 35 L/h (=approximately 20 L/h/m^2) in a PK study in patients with advanced solid tumors [12], which was consistent with estimates from population PK analysis (CL_{IV} = 38 L/h) [13]. Using the retrograde approach and the relative contribution of CYP3A (CYP3A4 fm= 82%, CYP3A5 fm= 8%) and additional hepatic metabolic clearance (HLM=10%) reported in vitro [14], the calculated unbound intrinsic clearance ($CL_{int,u}$) values for hepatic metabolic clearance of CYP3A4, CYP3A5, and HLM are 8.874 µL/min/pmol, 0.961 µL/min/pmol and 132.82 µl/min/10^6 hepatocytes, respectively. In cancer patients, approximately 8% of the IV dose was excreted unchanged in feces [15] and less than 5% of dose was excreted unchanged in urine [16]. The estimated renal clearance value in cancer patients was corrected by the GFR difference between cancer population and a healthy young subject to use as input. The mean contribution of each elimination pathway to total docetaxel clearance is as follows: around 78%, 1%, 9%, 8%, and 4% for CYP3A4, CYP3A5, additional hepatic metabolic clearance, biliary secretion, and renal clearance, respectively.

Table S4. **Physicochemical and pharmacokinetic input parameters for the PBPK model of docetaxel**

Parameter	Value	Reference
**Molecular weight (g/mol)**	807.89	Thai et al. (2015) [9]
**log P**	3.2	
**Compound type**	neutral	
**B/P**	0.68	Urien et al. (1996) [11]
**fu**	0.06	Predicted, Engels et al. (2004) [10]

**HSA KD (uM)**	137	Urien et al. (1996) [11]
**AGP KD (uM)**	6.9	
**% Bound to lipoprotein (CV)**	40 (15%)	
Distribution Model	Full PBPK Model	
**VSS (L/kg)**	2.37	Predicted (Method 2)
**Kp scalar**	1	
Elimination		
**Enzyme CYP3A4 CLint (µL/min/pmol)**	8.874	Back calculated from in vivo CL (fm based on Shou et al. (1998) [14])
**Enzyme CYP3A5 CLint (µL/min/pmol)**	0.961	
**HLM (µL/min/mg protein)**	132.82	
**CL bile (µL/min/million hepatocytes)**	42.34	Back calculated from in vivo CL (fe=8%, based on van Zuylen, et al. (2000) [15])
**CLR (L/h)**	2.65	Back calculated from in vivo CL (fe=5%, based on Clarke et al. (1999) [16])

HSA: Human serum albumin
AGP: α1- acid glycoprotein
KD: Dissociation constant of the drug-protein complex
Kp scalar: Scalar applied to all predicted tissue kp (tissue:plasma partition coefficients) value

2.2 Model Validation

The pharmacokinetic profiles of docetaxel (75 mg/m^2, 85 mg/m^2 and 100 mg/m^2) following administration of an IV infusion in the Pronk et al. [12] study served as the data set for internal validation, as the PBPK model utilized the PK information from this study for model development. The clinical PK data of docetaxel at doses 100 mg/m^2, reported in Brunsvig et al. and Rosing et al., were used as data sources for external validation to verify the performance of the PBPK model in predicting docetaxel pharmacokinetics [17, 18]. Clinical PK data of docetaxel at doses of 100 mg and 20-50 mg/m^2, reported in Oostendorp et al. and Hamberg et al. were also used to evaluate model performance and PK linearity [19, 20] . The assigned contribution of CYP3A4/5 used in the model was verified by the comparison of observed vs predicted interaction effect (C_{max}, AUC and CL ratio) of ketoconazole (a strong CYP3A4/5 inhibitor) on the PK of docetaxel [10, 21] . The detailed simulation condition for each study can be found in **Table S5**.

Table S5. Trial design for docetaxel model simulations

**Drug**	**Design**	**Reference (Observation)**
Docetaxel	Ten virtual trials with 33 subjects aged 33-74 years (24% female) receiving a single dose of 75,85, or 100 mg/m^2 docetaxel infused over 1 hour.	Pronk et al. (2000) [12]

	Ten virtual trials with 19 subjects aged 45-75 years (64% female) receiving a single dose of 100 mg/m^2 docetaxel infused over 1 hour.	Brunsvig et al. (2007) [17]
	Ten virtual trials with 24 subjects aged 33-73 years (96% female) receiving a single dose of 100 mg/m^2 docetaxel infused over 1 hour.	Rosing et al. (2000) [18]
	Ten virtual trials with 12 subjects aged 37-69 years (8% female) receiving a single dose of 100 mg docetaxel infused over 1 hour.	Oostendorp et al. (2009) [19]
	Ten virtual trials with 18 or 15 subjects aged 31-73 years (37% female) receiving a single dose of 20 or 50 mg/m^2 docetaxel infused over 1 hour.	Hamberg et al. (2015) [20]
	Ten virtual trials with 7 subjects aged 36 to 59 years (43% female) receiving a single dose of 10mg/m2 docetaxel infused over 1 hours in the absence or presence of ketoconazole (three 200-mg dose given 1 hour before the docetaxel infusion and 24 and 48 hours later.)	Engels et al. (2004) [10]
	Ten virtual trials with 7 subjects aged 44 to 69 years (14% female) receiving a single dose of 15mg/m2 docetaxel infused over 1 hours in the absence or presence of ketoconazole (400-mg dose every 8 hours, given 1 hour before the docetaxel infusion and up to 47 hours).	Engels et al. (2006) [21]

2.3. Results

The docetaxel model shown adequate predictive performance for PK across the dose range of 75-100 mg/m^2. The predicted Cmax, AUC and CL for doses of 75 mg/m^2, 85 mg/m^2 and 100 mg/m^2 were around 30% of the corresponding reported values in cancer patients [12]; however, no time-concentration data was reported in this study to allow comparison of the PK profile. Similarly, the predicted C_{max}, AUC and CL values reasonably agreed with the observed PK following the therapeutic dose of 100 mg/m^2 [17, 18](**Table S6a**). A representative predicted and observed ([17]) docetaxel plasma concentration-time profiles after a single 1-hour IV infusion of 100 mg/m^2 are shown in **Figure 3b**. In addition, the model adequately predicted the PK of docetaxel at a lower doses (100 mg and 20-50 mg/m^2), the predicted Cmax, AUC and CL were around 25% of the corresponding reported values in cancer patients [19, 20] (**Table S6a and Figure S2**).

The predicted and observed AUC ratios for docetaxel in the presence of ketoconazole are listed in **Table S6b**. The predicted values were in reasonable agreement with the observed data [10, 21].

Table S6a. **Comparison of clinically observed and PBPK predicted PK parameters of docetaxel**

Docetaxel		Cmax (µg/ml)	AUC_{inf} (µg/ml*h)	CL (L/h/m²)
**Pronk et al. (2000)** [12]	Predicted (n=330)	1.98 ± 0.22	2.94 ±0.38	26 ±4.18
**75mg/m²** **PK parameters reported as mean**	Observed (n=14)	2.47	2.98	27.4
	**Pred/Obs**	**0.80**	**0.99**	**0.95**
**Pronk et al. (2000)** [12]	Predicted (n=330)	2.24 ± 0.25	3.33 ±0.44	26 ±4.18
**85 mg/m²** **PK parameters reported as mean ± SD**	Observed (n=4)	2.64 ± 0.47	3.37 ± 0.97	27.7 ± 8.86
	**Pred/Obs**	**0.85**	**0.99**	**0.94**
**Pronk et al. (2000)** [12]	Predicted (n=330)	2.64 ± 0.3	3.92 ± 0.51	26 ±4.18
**100 mg/m²** **PK parameters reported as mean ± SD**	Observed (n=3)	3.73 ± 0.47	5.13 ± 0.51	19.7 ± 2.01
	**Pred/Obs**	**0.71**	**0.76**	**1.32**
**Brunsvig et al. (2007)** [17]	Predicted (n=190)	3236	4827	25.9
**100 mg/m²** **PK parameters reported as median and range** **Cmax (nM), AUC_{0-25h} (nMh)**		2514-4385	3799-6937	16.8-38
	Observed (n=19)	3737	5562	22.3
		2616-6949	3656-12790	10-34
	**Pred/Obs**	**0.87**	**0.87**	**1.16**
**Rosing et al. (2000)** [18]	Predicted (n=240)	2.50	3.75	27.05
**100 mg/m²** **PK parameters reported as mean and range**		2.2-3.26	2.9-5.47	17.8-39.68
	Observed (n=24)	2.60	3.10	34.80
		1.8-4	1.4-5.2	19.2-53.8
	**Pred/Obs**	**0.96**	**1.21**	**0.78**
**Oostendorp et al. (2009)** [19]	Predicted (n=120)	1.48±0.2	2.17±0.32	47
**100 mg** **PK parameters reported as mean and SD** **CL (L/h)**	Observed (n=12)	1.4±0.2	1.9±0.4	52.63
	**Pred/Obs**	**1.06**	**1.14**	**0.89**
**Hamberg et al. (2015)** [20]	Predicted (n=180)	0.53	0.79	25.66
**20 mg/m²** **PK parameters reported as mean and range**		0.42-0.7	0.62-1.09	16.77-37.64
	Observed (n=18)	0.694	0.857	25.4
		0.457-1.073	0.62-1.417	14.1-32.3
	**Pred/Obs**	**0.76**	**0.92**	**1.01**
**Hamberg et al. (2015)** [20]	Predicted (n=150)	1.34	1.98	25.67

**50 mg/m²** **PK parameters reported as mean and range**		1.06-1.76	1.55-2.73	16.87-35.94
	Observed (n=15)	1.415	1.602	31.5
		1.195-1.625	1.462-1.735	28.5-34.2
	**Pred/Obs**	**0.95**	**1.24**	**0.81**

Table S6b. Observed and predicted PK parameters ratios for docetaxel in absence and presence of ketoconazole

**Study**	**Inhibitor**		**Cmax Ratio (95% CI)**	**AUC Ratio (95% CI)**	**CL Ratio (95% CI)**
**Engels et al. (2004)** [10] **PK parameters reported as mean AUC$_{inf}$ Ratio;**	**Ketoconazole 200mg**	Predicted	1.28 (1.26-1.30)	1.50 (1.45-1.53)	0.68 (0.66-0.70)
		Observed	1.27 (0.72-1.81)	2.19 (1.39-2.99)	0.51 (0.36-0.65)
		**Pred/Obs**	**1.01**	**0.68**	**1.33**
**Engels et al. (2006)** [21] **PK parameters reported as mean, CI are not reported in this study**	**Ketoconazole 400mg**	Predicted	1.32	1.71	0.59
		Observed	1.02	2.08	0.50
		**Pred/Obs**	**1.30**	**0.82**	**1.17**

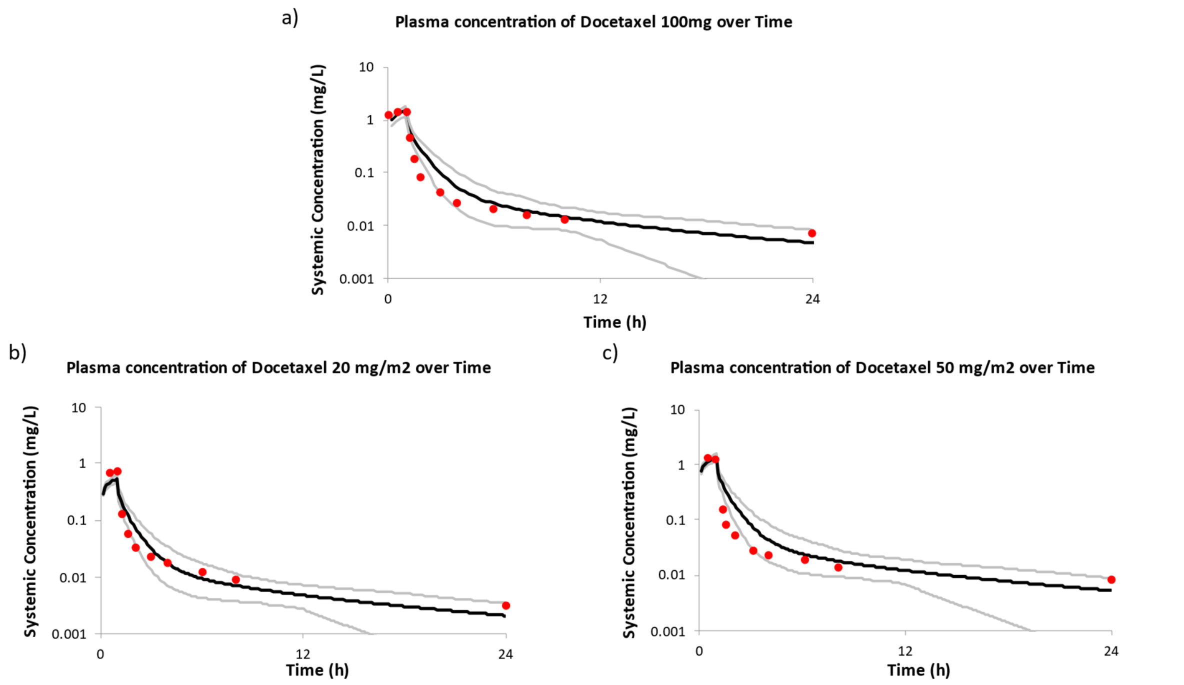

Figure S2. PBPK predicted vs observed plasma concentration-time profiles of docetaxel in cancer patients. (a) after 1-h infusion of 100 mg (Oostendorp et al. [19]); (b) after 1-h infusion of 20 mg/m^2 (Hamberg et al. [20]); (c) after 1-h infusion of 50 mg/m^2 (Hamberg et al. [20]). Red circles

represent the mean observed PK profile. Black and grey lines represent the population and 5th-95th percentiles of the predicted PK profiles, respectively.

3 Acalabrutinib

3.1 Drug model

The PBPK models of acalabrutinib and its metabolite ACP-5862 were based on the models described in Zhou et al. [22]. The physiochemical, absorption, distribution, metabolism, and elimination (ADME) input parameters are summarized in **Table S7**. A first-order absorption model was selected to describe the absorption process of acalabrutinib. The distribution was described using the full PBPK model with the Vss value of 0.21 L/kg optimized based on clinical PK data. The elimination component was kept consistent with the reference model. Acalabrutinib is predominantly metabolized by CYP3A4, accounting for approximately 79% of the metabolism, with a lesser contribution from glutathione at around 21%. The reported maximum rate of metabolism formation (Vmax) of 4.13 pmol/min/pmol and Km of 2.78 µM in CYP3A4 were incorporated in the model to describe the conversion of acalabrutinib to ACP-5862. The intrinsic clearance (CL_{int}) value of 8.14 µL/minutes/pmol was assigned for CYP3A4, to account for the remaining CYP3A4 metabolism. Additionally, human liver microsome clearance (CL_{int} of 289.5 µL/min/mg) was incorporated to represent glutathione metabolism, accounting for the remaining hepatic clearance of acalabrutinib. A renal clearance value of 1.33 L/h was used based on clinical data. The contribution of each elimination pathway to total acalabrutinib clearance for the single-dose of 100 mg is as follows: around 80%, 18% and 2% for CYP3A4, additional hepatic metabolic clearance and renal elimination, respectively. The competitive and time-dependent inhibition parameters against CYP2C8, CYP2C9, CYP2C19, and CYP3A enzymes for both acalabrutinib and ACP-5862 were incorporated in their respective models.

Table S7. **Physicochemical and pharmacokinetic input parameters for the PBPK model of acalabrutinib and its metabolite ACP-5862**

Parameter	Acalabrutinib	ACP-5862	Reference
**MW (g/mol)**	465.5	481.5	Zhou et al (2019)[22]
**Log P**	2.03	2.72	
**Compound type**	diprotic base	Diprotic base	
**pKa1/pKa2**	3.54,5.77	3.41, 4.49	
**B/P ratio**	0.787	0.65	
**fu**	0.026	0.013	
**Main plasma binding protein**	Human serum albumin	Human serum albumin	
Absorption model	First order		
**fa**	0.98		
**Ka (1/h)**	1.65		

**fu(gut)**	0.026	
**Tlag (hours)**	0.25	
**Q(gut) (L/h)**	12.33	
**Peff,man (x10^{-4} cm/second)**	4	
Distribution model	Full	Minimal
**Vss (L/kg)**	0.21	
**Kp scalar**	1	
**Vss (L/kg)**		0.36
**Vsac (L/kg)**		0.1
**kin (1/h)**		0.32
**kout (1/h)**		0.01
Elimination		
**Enzyme CYP3A4 CLint (µL/min/pmol)**	8.14	
**CYP3A4 Vmax (pmol/min/pmol)**	4.13	
**CYP3A4 Km (µM)**	2.78	
**Additional HLM (µl/min/mg)**	289.5	23.6
**CLR (L/h)**	1.33	0.3
Interaction		
**CYP2C8 Ki (µM)**	20.6	
**CYP2C9 Ki (µM)**	11.3	3.35
**CYP2C19 Ki (µM)**		8.5
**CYP3A4 Ki (µM)**	23.9	
**CYP3A4 KI (µM)**	10.1	
**CYP3A4 kinact (1/h)**	1.11	
**CYP2C8 kinact (1/h)**		0.72
**CYP2C8 KI (µM)**		4

Vsac (L/Kg): Volume of single adjusting compartment
Kp scalar: Scalar applied to all predicted tissue kp (tissue:plasma partition coefficients) value
Ki, inhibitory constant for reversible inhibition
KI, inhibitory constant for time-dependent inhibition

3.2 Model Validation

The model predictive values were compared with clinical study conducted in health volunteers and cancer patients across doses from 100 mg to 250 mg. In addition, results of the DDI studies of acalabrutinib with the strong CYP3A4 inhibitor itraconazole and the strong CYP3A4 inducer rifampicin were used to verify the assigned contribution of CYP3A4 in the PBPK model of acalabrutinib. The detailed simulation condition for each study can be found in **Table S8**.

Table S8. Trial design for acalabrutinib model simulations

Drug	Design	Reference (Observation)
Acalabrutinib	Ten virtual trials with 8 cancer subjects aged 44-84 years (26% female) receiving multiple doses of 100 mg acalabrutinib once daily.	Byrdet al. (2016)[23]
	Ten virtual trials with 28 cancer subjects aged 44-84 years (26% female) receiving multiple doses of 100 mg acalabrutinib twice daily.	
	Ten virtual trials with 7 cancer subjects aged 44-84 years (26% female) receiving multiple doses of 175 mg acalabrutinib once daily.	
	Ten virtual trials with 7 cancer subjects aged 44-84 years (26% female) receiving multiple doses of 250 mg acalabrutinib once daily.	
	Ten virtual trials with 16 healthy subjects aged 19 to 57 years (19% female) receiving a single oral dose of 50 mg acalabrutinib in the absence of itraconazole and on the last day of 6 days of dosing with itraconazole (200 mg BID)	Zhou et al. (2019) [22]
	Ten virtual trials with 24 healthy subjects aged 18 to 58 years (33% female) receiving a single oral dose of 100 mg acalabrutinib in the absence of rifampicin and on the last day of 9 days of dosing with rifampicin (600 mg QD)	

3.3 Results

The model was able to recover the PK profiles for acalabrutinib from 100 mg to 250 mg QD. For the therapeutic dose of 100 mg BID (day 8 PK), the predicted mean AUC was 1720 ng*h/mL and Cmax was 566 ng/mL. The model adequately described the observed mean AUC_{0-24} of acalabrutinib (1850 ng*h/mL), while underestimated the Cmax (827 ng/mL). However, a high variability was observed in this treatment group with a CV% of 102 [23]. Also, the underprediction of Cmax were not observed for other doses (or studies). The predictive performance for PK for the other doses is summarized in **Table S9a**.

The relative contribution of CYP3A4 to acalabrutinib metabolism was adequately assigned in the model as verified by the clinical DDI data with strong CYP3A modulators. In the presence of itraconazole 200 mg bid, the predicted vs observed changes in the Cmax ratio (CmaxR) and AUC ratio (AUCR) were 3.6-fold vs 3.9-fold (Cmax), and 5.0-fold vs 5.2-fold (AUC), respectively. With the coadministration of rifampicin, the predicted vs observed decreases in the CmaxR were 0.23-fold vs 0.32-fold, the predicted and observed decreases in the AUCR were 0.21-fold vs 0.23-fold (**Table S9b**).

Table S9a. Comparison of clinically observed and PBPK predicted PK parameters of acalabrutinib

		Cmax (ng/ml)	AUC_{0-24} (ng/ml*h)
**Byrd et al. (2016) [23] 100 mg QD**	Predicted (n=80)	580.37 ± 301.42	884.21 ± 542.62
	Observed (n=8)	529 ± 286	603 ± 179

**PK parameters reported as mean ± SD**	**Pred/Obs**	**1.10**	**1.47**
**Byrd et al. (2016) [23]<br>100 mg BID<br>PK parameters reported as mean ± SD**	Predicted (n=280)	565.92 ± 279.03	1719.71 ± 959.87
	Observed (n=28)	827 ± 841	1850 ± 1330
	**Pred/Obs**	**0.68**	**0.93**
**Byrd et al. (2016) [23]<br>175 mg QD<br>PK parameters reported as mean ± SD**	Predicted (n=70)	958.19 ± 482.04	1402.82 ± 735.92
	Observed (n=7)	805 ± 757	1160 ± 738
	**Pred/Obs**	**1.19**	**1.21**
**Byrd et al. (2016) [23]<br>250 mg QD<br>PK parameters reported as mean ± SD**	Predicted (n=70)	1383.5 ± 699.52	2031.37 ± 1075.44
	Observed (n=7)	1350 ± 933	2310 ± 1090
	**Pred/Obs**	**1.02**	**0.88**

Table S9b Comparison of clinically observed and PBPK predicted PK parameters of acalabrutinib in the absence and presence of itraconazole and rifampin

	**Cmax (ng/ml)**	**AUC_{0-24} (ng/ml*h)**	**Cmax (ng/ml)**	**AUC_{0-24} (ng/ml*h)**	**Cmax ratio (90% CI)**	**AUC ratio (90% CI)**
	**Control**		**+ itraconazole**			
**Predicted (n=160)**	220.76	293.77	789.79	1478.29	3.58 (3.46, 3.7)	5.03 (4.85, 5.22)
**Observed (n=16)**	166	239	649	1242	3.90 (3.2,4.76)	5.21 (4.6,5.89)
**Pred/Obs**	**1.33**	**1.23**	**1.22**	**1.19**	**0.92**	**0.97**
	**Control**		**+ rifampin**			
**Predicted (n=240)**	439.33	589.92	102.29	123.87	0.23 (0.22, 0.25)	0.21 (0.20, 0.22)
**Observed (n=24)**	450	641	142	150	0.32 (0.24, 0.42)	0.23 (0.19, 0.29)
**Pred/Obs**	**0.98**	**0.92**	**0.72**	**0.83**	**0.71**	**0.91**

Table S10. Comparison of clinically observed and model predicted clearance of paclitaxel of 175 mg/m² using the default healthy volunteers and cancer population models, and physiologically changes implemented in default cancer population model

Study	Population	fu	Albumin (g/L)	CYP3A4 Abundance (fm)	CYP2C8 Abundance (fm)	Renal GFR (fe)	CL (L/h)	
**Villalona-Calero et al. (1999) [5]**							Geo Mean	CV%
	Observation						21.20	19
	Sim-HV	0.06	43.75	138.03 (45.16%)	26.32 (42.10%)	107.76 (12.74%)	18.65	27
	Sim-Cancer	0.07	36.71	127.29 (44.91%)	27.24 (44.44%)	86.66 (10.65%)	18.08	31
**Kendra et al. (2015)[6]**							Median	Range
	Observation						22.30	(20.3, 39.1)
	Sim-HV	0.06	43.45	132.27 (40.66%)	33.46 (46.87%)	125.06 (12.47%)	23.67	(11.84,40.52)
	Sim-Cancer	0.07	35.99	133.78 (43.18%)	31.81 (46.55%)	84.07 (10.27%)	19.67	(9.25, 40.39)

CYP abundance expressed in pmol/mg-protein. Renal GFR expressed in mL/min/1.73m²
Fu: fraction unbound in plasma, fm: fraction of metabolism mediated by a specific enzyme. fe: fraction of parent drug excreted in urine

Table S11. Comparison of clinically observed and model predicted clearance of docetaxel of 100 mg/m² using the default healthy volunteers and cancer population models, and physiologically changes implemented in default cancer population model

Study	Population	fu	Albumin (g/L)	AGP (g/L)	% Bound to LPP	CYP3A4 Abundance (fm)	CYP3A5 Abundance (fm)	Renal GFR (fe)	CL (L/h/m²)	
									Median	Range
**Brunsvig et al. (2007) [17]**	Observation								22.3	(10, 34)
	Sim-HV	0.07	44.12	0.75	39.17	140.61 (77.69%)	23.4 (1.34%)	112.67 (4.89%)	27.94	(19.2, 39.2)
	Sim-Cancer	0.06	37.94	1.44	39.17	138.35 (77.07%)	20.33 (1.18%)	81.96 (3.90%)	25.91	(16.8, 38.1)
**Rosing et al. (2000) [18]**									Mean	Range
	Observation								34.8	(19.2, 53.8)
	Sim-HV	0.07	44.34	0.72	39.5	136.96 (75.90%)	25.11 (1.51)	128.34 (5.38%)	30.90	(21.7, 45.0)
	Sim-Cancer	0.06	38.7	1.39	39.5	137.43 (76.77%)	19.69 (1.14%)	81.71 (3.96%)	27.05	(17.8, 39.7)

CYP abundance expressed in pmol/mg-protein. Renal GFR expressed in mL/min/1.73m²
AGP: alpha(1)-Acid glycoprotein; fu: fraction unbound in plasma; fm: fraction of metabolism mediated by a specific enzyme; fe: fraction of parent drug excreted in urine.

Table S12. **Comparison of observed and predicted PK parameter of paclitaxel 175 mg/m² after 3-hr infusion in pregnant patients with cancer, and physiologically changes implemented in the default pregnancy or modified pregnancy population models**

Study	Population Model	fu	Albumin (g/L)	CYP3A4 Abundance (fm)	CYP2C8 Abundance (fm)	Renal GFR (fe)	Cmax (mg/L)		AUC (mg*h/L)		CL (L/h)	
van Hasselt et al. (2014) [2]								Pred/Obs		Pred/Obs		Pred/Obs
	Observation (n=5, GW=23)						*2.18*		*8.68*		*35.72*	
	Sim-HV	0.05	44.66	128.72 (48.96%)	28.4 (37.15%)	148.03 (13.89%)	3.1		14.57		22.38	
	Sim-Pregnancy (GW=32)	0.06	38.94	202.28 (47.53%)	26.46 (25.18%)	198.22 (27.29%)	*2.62*	*1.20*	*11.83*	*1.36*	*27.65*	*0.77*
	Sim-Pregnancy CYP2C8 2.5-fold increase (GW=32)	0.06	38.94	202.28 (43.02%)	66.16 (33.26%)	198.22 (23.71%)	*2.34*	*1.07*	*9.82*	*1.13*	*33.17*	*0.93*
Janssen et al. (2021) [3]	Observation (n=20, GW=31)						*3.28*		*8.04*		*39.2*	
	Sim-HV	0.05	44.96	135.20 (47.93%)	26.17 (38.25%)	145.94 (13.88%)	3.15		14.49		22.50	
	Sim-Pregnancy (GW=32)	0.07	36.42	255.48 (49.41%)	24.8 (23.43%)	195.54 (27.16%)	*2.46*	*0.75*	*11.05*	*1.38*	*30.33*	*0.77*
	Sim-Pregnancy CYP2C8 2.5-fold increase (GW=32)	0.07	36.42	255.48 (34.93%)	62 (41.41%)	195.54 (23.65%)	*2.24*	*0.68*	*9.46*	*1.18*	*35.35*	*0.90*

CYP abundance expressed in pmol/mg-protein. Renal GFR expressed in mL/min/1.73m²

fu: fraction unbound in plasma; fm: fraction of metabolism mediated by a specific enzyme; fe: fraction of parent drug excreted in urine.

Table S13. **Comparison of observed and predicted PK parameter of docetaxel 100 mg/m² after 1-hr infusion in pregnant patients with cancer, and physiologically changes implemented in the default pregnancy population model**

	Population Model	fu	Albumin (g/L)	AGP (g/L)	% Bound to LPP	CYP3A4 Abundance (fm)	CYP3A5 Abundance (fm)	Renal GFR (fe)	Cmax (mg/L)		AUC (mg*h/L)		CL (L/h)	
									Pred	Pred/Obs	Pred	Pred/Obs	Pred	Pred/Obs
van Hasselt et al. (2014) [2]	Observation (n=3, GW=32)								*1.71*		*2.25*		*79.88*	
	Sim-HV	0.08	44.05	0.72	38.2	135.42 (75.23%)	26.8 (1.55%)	150.74 (5.68%)	2.02		2.89		59.91	
	Sim-Pregnancy (GW=32)	0.09	35.07	0.6	38.2	263.2 (82.39%)	29.22 (1.07%)	193.96 (6.65%)	*1.85*	*1.08*	*2.80*	*1.24*	*65.29*	*0.82*
Janssen et al. (2021) [3]	Observation (n=9, GW=32)								*1.73*		*2.38*		*75.66*	
	Sim-HV	0.07	44.54	0.72	39.18	129.55 (73.86%)	24.56 (1.52%)	148.59 (5.75%)	2.05		2.95		58.42	

Sim-Pregnancy (GW=32)	0.09	35.82	0.59	39.18	250.61 (81.39%)	32.04 (1.21%)	200.1 (6.89%)	*1.86*	*1.08*	*2.82*	*1.18*	*65.05*	*0.86*

CYP abundance expressed in pmol/mg-protein. Renal GFR expressed in mL/min/1.73m².

AGP: alpha(1)-Acid glycoprotein; fu: fraction unbound in plasma; fm: fraction of metabolism mediated by a specific enzyme; fe: fraction of parent drug excreted in urine.

Table S14. **Comparison of pharmacokinetics parameters of paclitaxel and docetaxel between nonpregnant and pregnant cancer patients**

		Cmax (mg/L)			AUC (mg*h/L)			CL (L/h)		
**Paclitaxel 175 mg/m²**		**Pred**	**Obs**	**Pred/Obs**	**Pred**	**Obs**	**Pred/Obs**	**Pred**	**Obs**	**Pred/Obs**
**Villalona-Calero et al. (1999) [5]**	***Non-pregnant with Cancer***	3.77	3.98	0.95	17.48	16.05	1.09	18.15	21.2	0.86
**Van Hasselt et al. (2014) [2]**	***Pregnant with Cancer***	2.34	2.18	1.07	9.82	8.68	1.13	33.17	35.72	0.93
	Pregnancy/Non-pregnant with Cancer	**0.62**	**0.55**		**0.56**	**0.54**		**1.83**	**1.68**	

**Docetaxel 100 mg/m²**		**Pred**	**Obs**	**Pred/Obs**	**Pred**	**Obs**	**Pred/Obs**	**Pred**	**Obs**	**Pred/Obs**
**Rosing et al. (2000) [18]**	***Non-pregnant with Cancer***	2.51	2.6	0.97	3.53	3.1	1.14	28.8	34.8	0.83
**van Hasselt et al. (2014) [2]**	***Pregnant with Cancer***	1.85	1.708	1.08	2.76	2.253	1.23	66.33	79.88	0.83
	Pregnancy/Non-pregnant with Cancer	**0.74**	**0.66**		**0.78**	**0.73**		**2.30**	**2.30**	

References

1. Mendes, M.S., et al., *A physiologically based pharmacokinetic - pharmacodynamic modelling approach to predict incidence of neutropenia as a result of drug-drug interactions of paclitaxel in cancer patients.* Eur J Pharm Sci, 2020. **150**: p. 105355.
2. van Hasselt, J.G.C., et al., *Optimizing anticancer drug treatment in pregnant cancer patients: pharmacokinetic analysis of gestation-induced changes for doxorubicin, epirubicin, docetaxel and paclitaxel.* Ann Oncol, 2014. **25**(10): p. 2059-2065.
3. Janssen, J.M., et al., *Population Pharmacokinetics of Docetaxel, Paclitaxel, Doxorubicin and Epirubicin in Pregnant Women with Cancer: A Study from the International Network of Cancer, Infertility and Pregnancy (INCIP).* Clin Pharmacokinet, 2021. **60**(6): p. 775-784.
4. Walle, T., et al., *Taxol metabolism and disposition in cancer patients.* Drug Metab Dispos, 1995. **23**(4): p. 506-12.
5. Villalona-Calero, M.A., et al., *Phase I and pharmacokinetic study of the oral fluoropyrimidine capecitabine in combination with paclitaxel in patients with advanced solid malignancies.* J Clin Oncol, 1999. **17**(6): p. 1915-25.
6. Kendra, K.L., et al., *A multicenter phase I study of pazopanib in combination with paclitaxel in first-line treatment of patients with advanced solid tumors.* Mol Cancer Ther, 2015. **14**(2): p. 461-9.
7. Brouwer, E., et al., *Measurement of fraction unbound paclitaxel in human plasma.* Drug Metab Dispos, 2000. **28**(10): p. 1141-5.
8. Berg, S.L., et al., *Effect of R-verapamil on the pharmacokinetics of paclitaxel in women with breast cancer.* J Clin Oncol, 1995. **13**(8): p. 2039-42.
9. Thai, H.T., et al., *Optimizing pharmacokinetic bridging studies in paediatric oncology using physiologically-based pharmacokinetic modelling: application to docetaxel.* Br J Clin Pharmacol, 2015. **80**(3): p. 534-47.
10. Engels, F.K., et al., *Effect of cytochrome P450 3A4 inhibition on the pharmacokinetics of docetaxel.* Clin Pharmacol Ther, 2004. **75**(5): p. 448-54.
11. Urien, S., et al., *Docetaxel serum protein binding with high affinity to alpha 1-acid glycoprotein.* Invest New Drugs, 1996. **14**(2): p. 147-51.
12. Pronk, L.C., et al., *A phase I and pharmacokinetic study of the combination of capecitabine and docetaxel in patients with advanced solid tumours.* Br J Cancer, 2000. **83**(1): p. 22-9.
13. Bruno, R., et al., *A population pharmacokinetic model for docetaxel (Taxotere): model building and validation.* J Pharmacokinet Biopharm, 1996. **24**(2): p. 153-72.
14. Shou, M., et al., *Role of human cytochrome P450 3A4 and 3A5 in the metabolism of taxotere and its derivatives: enzyme specificity, interindividual distribution and metabolic contribution in human liver.* Pharmacogenetics, 1998. **8**(5): p. 391-401.
15. van Zuylen, L., et al., *Role of intestinal P-glycoprotein in the plasma and fecal disposition of docetaxel in humans.* Clin Cancer Res, 2000. **6**(7): p. 2598-603.
16. Clarke, S.J. and L.P. Rivory, *Clinical pharmacokinetics of docetaxel.* Clin Pharmacokinet, 1999. **36**(2): p. 99-114.
17. Brunsvig, P.F., et al., *Pharmacokinetic analysis of two different docetaxel dose levels in patients with non-small cell lung cancer treated with docetaxel as monotherapy or with concurrent radiotherapy.* BMC Cancer, 2007. **7**: p. 197.

18. Rosing, H., et al., *Pharmacokinetics and metabolism of docetaxel administered as a 1-h intravenous infusion.* Cancer Chemother Pharmacol, 2000. **45**(3): p. 213-8.
19. Oostendorp, R.L., et al., *Coadministration of ritonavir strongly enhances the apparent oral bioavailability of docetaxel in patients with solid tumors.* Clin Cancer Res, 2009. **15**(12): p. 4228-33.
20. Hamberg, P., et al., *Impact of pazopanib on docetaxel exposure: results of a phase I combination study with two different docetaxel schedules.* Cancer Chemother Pharmacol, 2015. **75**(2): p. 365-71.
21. Engels, F.K., et al., *Influence of high-dose ketoconazole on the pharmacokinetics of docetaxel.* Cancer Biol Ther, 2006. **5**(7): p. 833-9.
22. Zhou, D., et al., *Evaluation of the Drug-Drug Interaction Potential of Acalabrutinib and Its Active Metabolite, ACP-5862, Using a Physiologically-Based Pharmacokinetic Modeling Approach.* CPT Pharmacometrics Syst Pharmacol, 2019. **8**(7): p. 489-499.
23. Byrd, J.C., et al., *Acalabrutinib (ACP-196) in Relapsed Chronic Lymphocytic Leukemia.* N Engl J Med, 2016. **374**(4): p. 323-32.
